# Metropolitan Hospital Sunday Fund

**Published:** 1895-11-09

**Authors:** 


### METROPOLITAN HOSPITAL SUNDAY FUND.

### PARTICULARS OF THE SECOND DISTRIBUTION FOR 1895.

Total Sum Received, £61,500.

The following is the second report of the Committee of Distribution, 1895, adopted by the council on the 7th inst, the Lord Mayor (Sir Joseph Renals, Bart.) in the chair. The Lord Mayor having received additional contributions, amounting to nearly £16,000, since the distribution made to the hospitals and dispensaries, &c., about three months ago, a special meeting of the committee was convened to consider the desirability of making a second distribution this year, when it was unanimously resolved to recommend the council to sanction the amounts set forth in the annexed appendix. The awards recommended are all calculated precisely on the same bases as the sums approved by the council and paid in August last. (See The Hospital, August 3rd, 1895, page 315.) The usual 5 per cent. on the total amount of the Fund for the year is reserved for surgical appliances, as provided by Law 12. The account of the meeting will appear in our next issue:—

Twenty-seven General Hospitals.

Charing Cross Hospital; £393 15s.; French Hospital, £153 15s.; German Hospital, £226 17s. 6d.; Great Northern Central Hospital, £140 12s. 6d.; Guy's Hospital, £262 10s.; Hampstead Hospital, £41 5s.; Italian Hospital, £30; King's College Hospital, £600; London Hospital, £1,406 5s.; London Homœopathic Hospital, £52 10s.; London Temperance Hospital. £262 10s.; Metropolitan Hospital, £187 10s.; Miller Hospital and Royal Kent Dispensary, £97 10s.; North-West London Hospital, £142 10s.; Poplar Hospital, £151 17s. 6d.; Queen's Jubilee Hospital, nil; Royal Free Hospital, £337 10s.; St. George's Hospital, £562 10s.; SS. John and Elizabeth Hospital, £37 10s.; St. Mary's Hospital, £750; Seamen's Hospital Society, £345; The Middlesex Hospital. £806 5s.; Training Hospital, Tottenham, nil; University College Hospital, £487 10s.; West Ham Hospital, £101 5s.; West London Hospital, £225; Westminster Hospital, £431 5s.

Special Hospitals.

Five Chest Hospitals.—City of London Hospital for Diseases of the Chest, Victoria Park, £382 10s.; Hospital for Consumption, Brompton, £603 15s.; North London Consumption Hospital, Hampstead, £135; Royal Hospital for Diseases of the Chest, City Road, £150; Royal National Hospital for Consumption (Ventnor), £112 10s.

Thirteen Children's Hospitals.—Alexandra Hospital for Hip Disease, Bloomsbury, W.C., £67 10s.; Barnet Children's Home and Hospital, £15; Belgrave Hospital for Children, S.W., £60; Cheyne Hospital for Incurable Children, S.W., £56 5s.; East London Hospital for Children, Shadwell, E., £187 10s.; Evelina Hospital for Sick Children, Southwark, S.E., £168 15s; Home for Incurable Children, Maida Vale, W., £26 5s.; Home for Sick Children, Sydenham, S.E., £56 5s.; Hospital for Sick Children, Great Ormond Street, W.C., £300; North-Eastern Hospital for Children, Hackney Road, N.E., £112 10s.; Paddington Green Hospital for Children, W., £56 5s.; Victoria Hospital for Children, King's Road, Chelsea, S.W., £225; "The Vine," Seven oaks, £11 5s.

Six Lying-in Hospitals.—British Lying-in Hospital, Endell Street, W.C., £15; City of London Lying-in Hospital, City Road, E.C., £30; Clapham Maternity Hospital, S.W., £7 10s.; East end Mothers' Home, E., £22 10s.; General Lying-in Hospital, Lambeth, S E., £22 10s.; Queen Charlotte's Lying-in Hospital, Marylebone Road, W., £135.

Six Hospitals for Women.—Chelsea Hospital for Women, S.W., £75; Hospital for Women, Soho Square, W., £153 15s.; Grosvenor Hospital for Women and Children, Vincent Square, S.W., £41 5s.; New Hospital for Women, Euston Road, W.C., £75; Royal Hospital for Children and Women, Lambeth, S.E., £93 15s.; Samaritan Free Hospital, Lower Seymour Street, W., £213 15s.

Twenty-eight Other Special Hospitals.

Cancer Hospital, Brompton, S.W., £262 10s.; London Fever Hospital, Islington, N.; £225; Gordon Hospital for Fistula, Vauxhall Bridge Road, S.W., £15; St. Mark's Hospital for Fistula, City Road, E.C., £22 10s.; National Hospital for the Diseases of the Heart and Paralysis, Soho Square, W., £33 15s.; Female Lock Hospital, Harrow Road, W., £67 10s.; Male Lock Hospital, Soho, W., nil; Hospital for Epilepsy, Paralysis, and other Diseases of the Nervous System, Regent's Park, N.W., £22 10s.; National Hospital for the Paralysed and Epileptic, W.C., £318 15s.; West End Hospital for Diseases of the Nervous System, Welbeck Street, W., £30; Central London Ophthalmic Hospital, Gray's Inn Road, W.C., £13 10s.; Royal Eye Hospital, St. George's Circus, S.E., £56 5s.; Royal London Ophthalmic Hospital, Moorfields, E.C., £210; Royal Westminster Ophthalmic Hospital, Charing Cross, W.C., £37 10s.; Western Ophthalmic Hospital, Marylebone Road, W., £12 7s. 6d.; City Orthopædic Hospital, Hatton Garden, E.C., £30; National Orthopædic Hospital, Great Portland Street, W., £18; Royal Orthopædic Hospital, Oxford Street, W., £30; Royal Sea-Bathing Infirmary, Margate, £150; Hospital for Diseases of the Skin, Stamford Street, S.E., £7 10s.; Western Skin Hospital, nil; St. Peter's Hospital for Stone, Covent Garden, W.C., £18 15s.; Central London Throat and Ear Hospital, Gray's Inn Road, W.C., £20 12s. 6d.; Hospital for Diseases of the Throat, Golden Square, W., £11 5s.; Royal Ear Hospital, Frith Street, W., £2 5s.; Phillips Memorial Homœopathic Hospital, Bromley, Kent, £13 2s. 6d.; The Dental Hospital of London, Leicester Square, W.C., £45; National Dental Hospital, 149, Great Portland Street, W., £31 17s. 6d.

Twenty-two Convalescent Hospitals.

Metropolitan Convalescent Institution, Sackville Street, W., £243 15s.; Bexhill Convalescent Home, Sackville Street, W., £112 10s.; All Saints' Convalescent Hospital, Eastbourne, £206 5s.; Mrs. Gladstone's Convalescent Home, Woodford, E., £37 10s.; Hahnemann Convalescent Home, Bournemouth, £15; Hanwell Convalescent Home, W., £7 10s.; Herbert Convalescent Home, Bournemouth, £11 5s.; Herne Bay, Baldwin-Browne Convalescent Home, £9 7s. 6d.; Homœopathic Convalescent Home, Eastbourne, £3 15s.; King's College Hospital Convalescent Home, Hemel Hempstead, £29 11s.; Mrs. Kitto's Convalescent Home, Reigate, £33 15s.; London and Brighton Female Convalescent Home, £20 12s. 6d.; Mary Wardell Convalescent Home for Scarlet Fever, £37 17s. 6d.; Morley House Convalescent Home for Working Men, £78 15s.; Princess Frederica's Convalescent Home, East Molesey, £5 12s. 6d.; St. Andrew's Convalescent Hospital, Clewer, £63 15s.; St. Andrew's Convalescent Home, Folkestone, £93 15s.; St. John's Home for Convalescent and Crippled Children, Brighton, £22 10s.; St. Joseph's Convalescent Home, Bournemouth, £18 15s.; St. Leonards-on-Sea Convalescent Home for Poor Children, £48 15s.; St. Michael's Convalescent Home, Westgate-on-Sea, £15; Seaside Convalescent Hospital, Seaford, £56 5s.

Thirteen Cottage Hospitals.

Beckenham Cottage Hospital, £18 15s.; Blackheath and Charlton Cottage Hospital, £26 5s.; Bromley, Kent, Cottage Hospital, £28 2s. 6d.; Chislehurst, Sidcup, and Cray Valley Cottage Hospital, £15; Eltham Cottage Hospital, £11 5s.; Enfield Cottage Hospital, £15; Epsom and Ewell Cottage Hospital, £26 5s.; Hounslow Cottage Hospital, £13 2s. 6d.; Reigate and Redhill Cottage Hospital, £20 12s. 6d.; Sidcup Cottage Hospital, £11 5s.; Warley Cottage Hospital, Essex, £3; Wimbledon Cottage Hospital, £15; Woolwich and Plumstead Cottage Hospital, £11 5s.

Five Institutions

Establishment for Gentlewomen, Harley Street, £41 5s.; National Sanatorium for Consumption, Bournemouth, £22 10s.; Invalid Asylum, Stoke Newington, £20 12s. 6d.; Firs Home, Bournemouth, £15; St. Catherine's Home, Ventnor, £13 2s. 6d.

Fifty-four Dispensaries.

Battersea Provident Dispensary, £26 5s.; Bloomsbury Provident Dispensary, £4 10s.; Brixton, &c., Dispensary, £22 10s.; Brompton Provident Dispensary, £10 2s. 6d.; Camberwell Provident Dispensary, £37 10s; Camden Provident Dispensary, £5 5s.; Chelsea, Brompton, and Belgrave Dispensary, £17 12s. 6d.; Chelsea Provident Dispensary, £4 17s. 6d.; City Dispensary, £32 12s. 6d.; City of London and East London Dispensary, £7 10s.; Clapham General and Provident Dispensary, £13 2s. 6d.; Deptford Medical Mission, £5 12s. 6d; Eastern Dispensary, £14 5s.; East Dulwich Provident Dispensary, £7 10s.; Farringdon General Dispensary, £16 17s. 6d.; Finsbury Dispensary, £20 12s. 6d.; Forest Hill Dispensary, £14 5s.; Hackney Provident Dispensary, £2 12s. 6d.; Hampstead Provident Dispensary, £16 2s. 6d.; Holloway and North Islington Dispensary, £24 7s. 6d.; Islington Dispensary, £16 17s. 6d.; Kensal Town Provident Dispensary, £3 7s. 6d.; Kensington Dispensary, £26 5s.; Kilburn, Maida Vale, and St. John's Wood Dispensary, £16 2s. 6d.; Kilburn Provident Medical Institution, £11 5s.; London Dispensary, £6 15s.; London Medical Mission, Endell Street, £26 5s.; Marylebone Medical Mission, nil; Metropolitan Dispensary, £16 17s. 6d.; Notting Hill Dispensary and Maternity (Provident), £8 5s.; Paddington Provident Dispensary, £15; Pimlico Provident Dispensary (Lupus Street), £6 15s.; Portland Town Dispensary, £7 10s.; Portobello Road Provident Dispensary, £2 12s. 6d.; Public Dispensary, £18 15s.; Queen Adelaide's Dispensary, £15; Royal General Dispensary, £15; Royal Pimlico Prnvident Dispensary, £16 17s. 6d.; Royal South London Dispensary, £22 10s.; St. George's and St. James's Disp nsary, £19 10s.; St. George's, Hanover Square, Provident Dispensary, £16 2s. 6d.; St. John's Wood and Portland Town Provident Dispensary. £11 5s.; St. Marylebone General Dispensary, £16 17s. 6d.; St. Pancras and Northern Dispensary, £16 17s. 6d; South Lambeth, Stockwell, and North Brixton Dispensary, £16 17s. 6d.; Stamford Hill, Stoke Newington, &c., Dispensary, £20 12s 6d.; Tower Hamlets Dispensary, £16 2s. 6d.; Walworth Provident Dispensary, £4 10s.; Wandsworth Common Provident Dispensary, £3 7s. 6d.; Westbourne Provident Dispensary and Maternity, £5 12s. 6d.; Western Dispensary, £22 10s.; Western General Dispensary, £50 12s. 6d.; Westminster General Dispensary, £11 12s. 6d.; Whitechapel Provident Dispensary, £9.

